# Unfused Crossed Renal Ectopia With Aberrant Vasculature: A Case Report

**DOI:** 10.1155/criu/1244129

**Published:** 2026-03-06

**Authors:** Adam J. Ferguson, Matthew Janiga

**Affiliations:** ^1^ Surgery Department, Sutter Davis Hospital, Davis, California, USA; ^2^ Urology, Sutter Medical Group, Auburn, California, USA, suttermedicalfoundation.org

## Abstract

An 80‐year‐old man presenting with gross hematuria was diagnosed with muscle‐invasive bladder cancer. Ten years prior, based on an ultrasound performed for chronic renal insufficiency, he was told he had a congenitally absent left kidney. Office cystoscopy showed a large bladder tumor. Following cystoscopy, he underwent a CT‐urogram. This study showed that he, in fact, had left‐to‐right unfused crossed renal ectopia. Unfused crossed renal ectopia is an extremely rare abnormality that is usually asymptomatic and typically incidentally diagnosed. Crossed renal ectopia is a result of improper migration of the metanephric blastema and ureteric bud development resulting in a contralateral ectopic kidney with aberrant vascularization. The ureter crosses the midline and inserts into the orthotopic side of the bladder. Special consideration of the vascularization of unfused crossed ectopic kidneys is necessary prior to surgery or radiation therapy.

## 1. Introduction

Unfused crossed renal ectopia is 10 times rarer than fused renal ectopia (1: 75,000 vs. 1:7500) [1]. Left‐to‐right crossed renal ectopia is 2–3 times as likely as right‐to‐left, and men are 1.4–2 times as likely as women to have crossed renal ectopia [2, 3]. Crossed renal ectopia is thought to be a result of the failure of the metanephric blastema and ureteric bud to ascend from the cloaca to the retroperitoneal area and the aberrant migration of the metanephric blastema and ureteric bud across the midline during the first 4–8 weeks of development [4]. The resultant kidney resides on the contralateral side, typically proximal to the midline and caudal to the orthotopic kidney, residing within or near the pelvis or false pelvis. The ureter crosses the midline and inserts into the orthotopic side of the bladder [2, 5]. Special consideration should be given to the vascularization of the ectopic kidney. The ectopic renal artery may branch from atypical positions on the abdominal aorta, the common iliac artery, or median sacral artery [2]. As in this presented case, unfused cross‐renal ectopia is commonly misdiagnosed as unilateral renal agenesis and is commonly diagnosed incidentally [2].

## 2. Case Presentation

**Figure 1:**
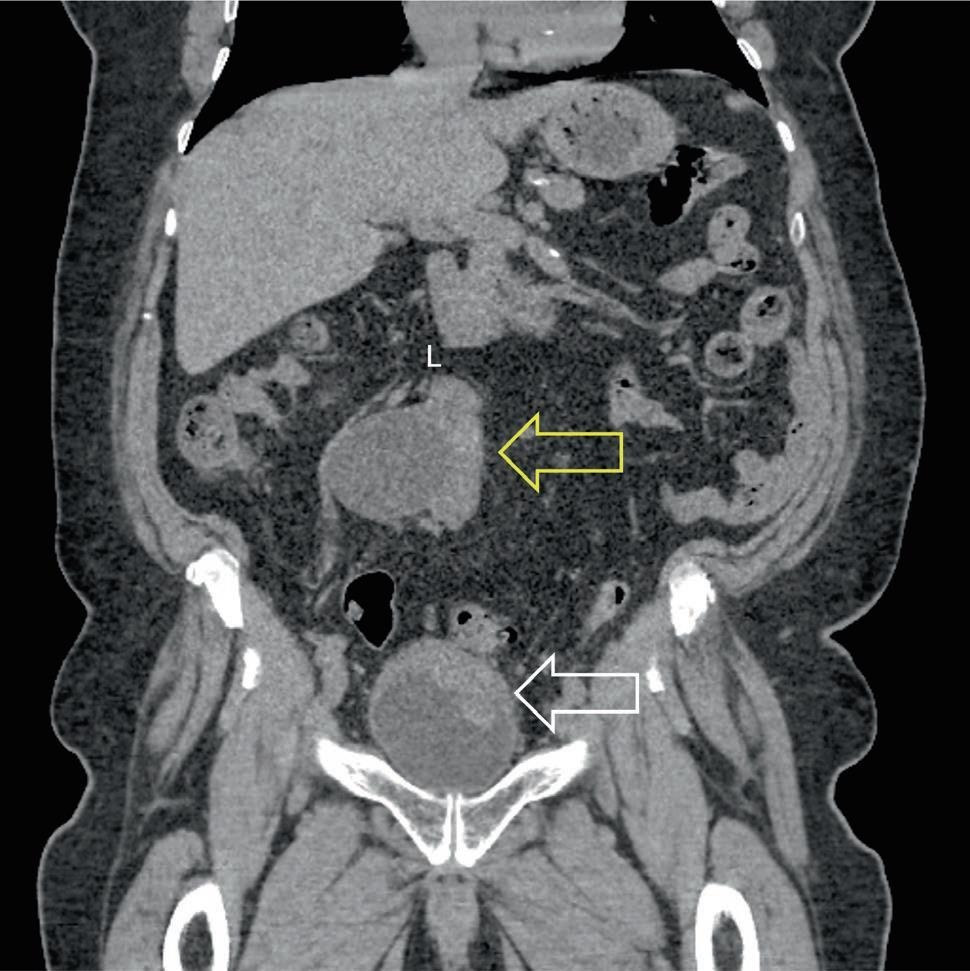
Coronal view of noncontrast CT‐urogram. White arrow indicates muscle‐invasive bladder cancer. Yellow arrow indicates left‐to‐right unfused crossed ectopic kidney.

An 80‐year‐old male who is a former smoker with a history of coronary artery disease, chronic renal insufficiency, high cholesterol, and hypertension presented with gross hematuria. His creatinine level was 1.25 mg/dL, and estimated glomerular filtration rate was 58 mL/min/1.73m^2^. Ten years prior, he was misdiagnosed with a solitary right kidney with a congenital absence of the left kidney after an ultrasound ordered due to his renal insufficiency. Flexible office cystoscopy showed a large bladder tumor and subsequent CT‐urogram showed nonmetastatic muscle‐invasive bladder cancer (Figure 1). The CT‐urogram showed that the previous diagnosis of solitary right kidney was incorrect. He had left‐to‐right unfused crossed renal ectopia (Figure 1).

**Figure 2:**
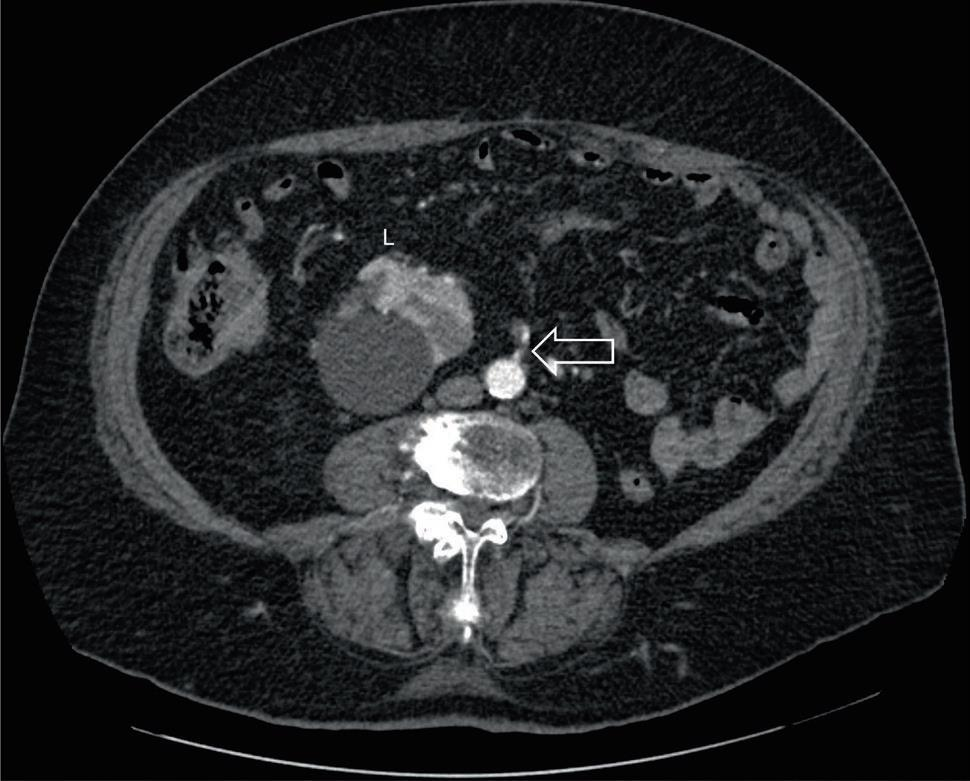
Axial view of arterial phase CT‐urogram. White arrow indicates the left main renal artery originating from the left anterior wall of the abdominal aorta. Left ectopic kidney is marked by white “L”. The left kidney is malrotated with the hilum facing right and posterior, away from the midline. A large, benign renal cyst lies lateral to the left kidney′s hilum.

Careful analysis of the CT‐urogram shows unusual vasculature. The orthotopic right kidney has duplicated right renal arteries. The main renal artery originates normally from the aorta. The right renal vein is unremarkable with normal anatomy. The crossed‐ectopic left kidney′s hilum faced right and posterior, and the kidney resides above the pelvic brim in the false pelvis (Figure 1). The main left renal artery originates off the left anterior wall of the abdominal aorta caudal to the inferior mesenteric artery and crosses the midline anterior to the aorta and ran anterior to the left kidney (Figure 2). Adding to the irregularity of the renal vasculature, the lower pole of the left crossed‐ectopic kidney and the lower pole of the normally positioned right kidney are both supplied with arterial blood from a common artery branching off the right side of the aorta, just inferior to the main artery supplying the ectopic left kidney (Figure 3). The left renal vein is short and inserts onto the right anterior surface of the vena cava (Figure 3).

Figure 3Axial view of the arterial phase CT‐urogram moving cranially from (a) to (e). This sequence follows the arterial blood supply originating from the aorta and ascending to the kidneys. White arrow indicates the common artery which supplies the lower poles of the left and right kidneys. The yellow arrow indicates the branch off the common artery supplying the left kidney. The red arrow indicates the branch off the common artery supplying the right artery. The green arrow indicates the left renal vein. The left and right kidneys are marked with “L” and “R,” respectively.

**Figure:**
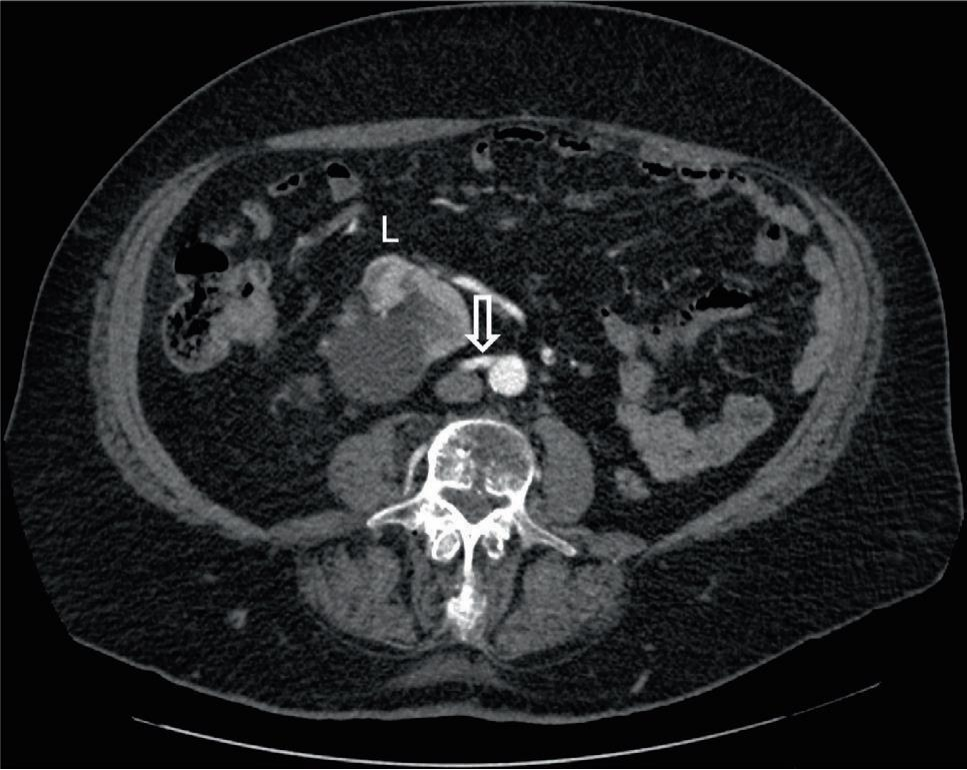
(a)

**Figure:**
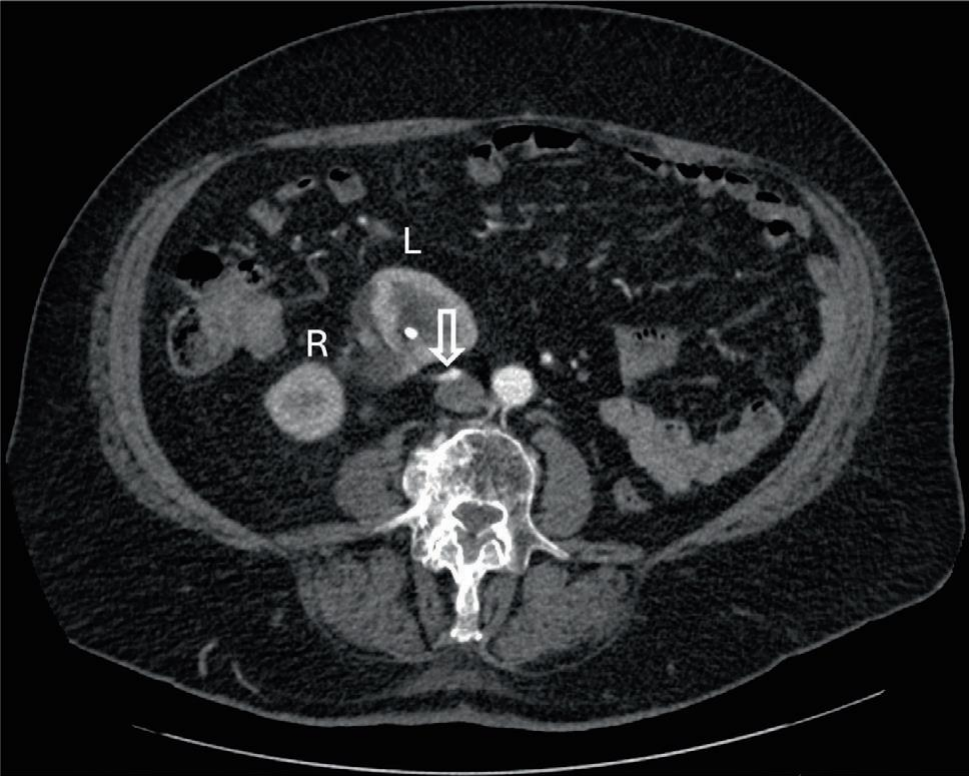
(b)

**Figure:**
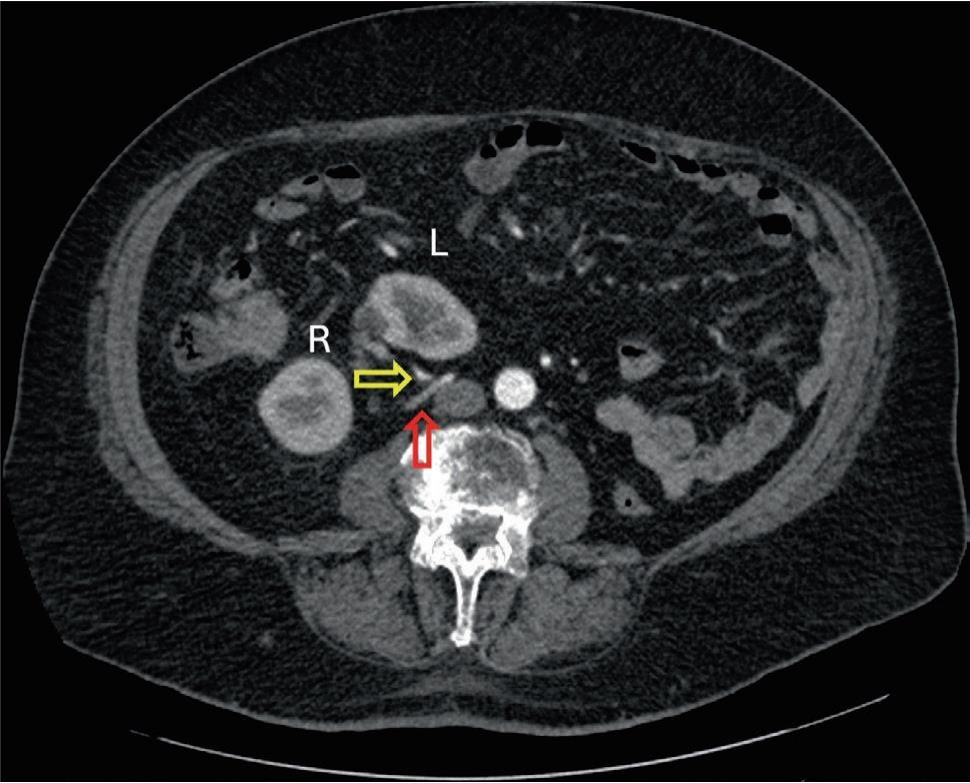
(c)

**Figure:**
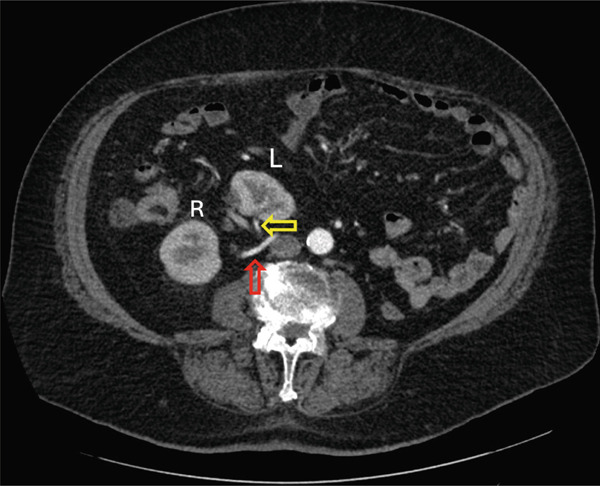
(d)

**Figure:**
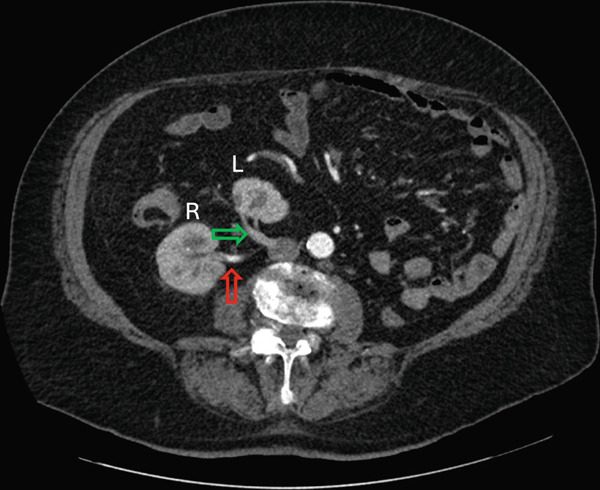
(e)

Retrograde pyelograms showed orthotopic right and left ureteral orifices (Figure 4). There are no upper tract filling defects. The left renal cist noted in Figure 2 is appreciated in the retrograde pyelogram (Figure 4). The left ureter crosses the midline to the ectopic left kidney (Figure 4). Transurethral resection of bladder tumor (TURBT) shows muscle‐invasive micropapillary variant transitional cell cancer. The location of the ectopic kidney is high enough in the false pelvis to not interfere with planned chemoradiation.

Figure 4Retrograde pyelogram of (a) the left ureter inserted into the orthotopic position on the left side of the bladder and (b) the left kidney and left and right ureters.

**Figure:**
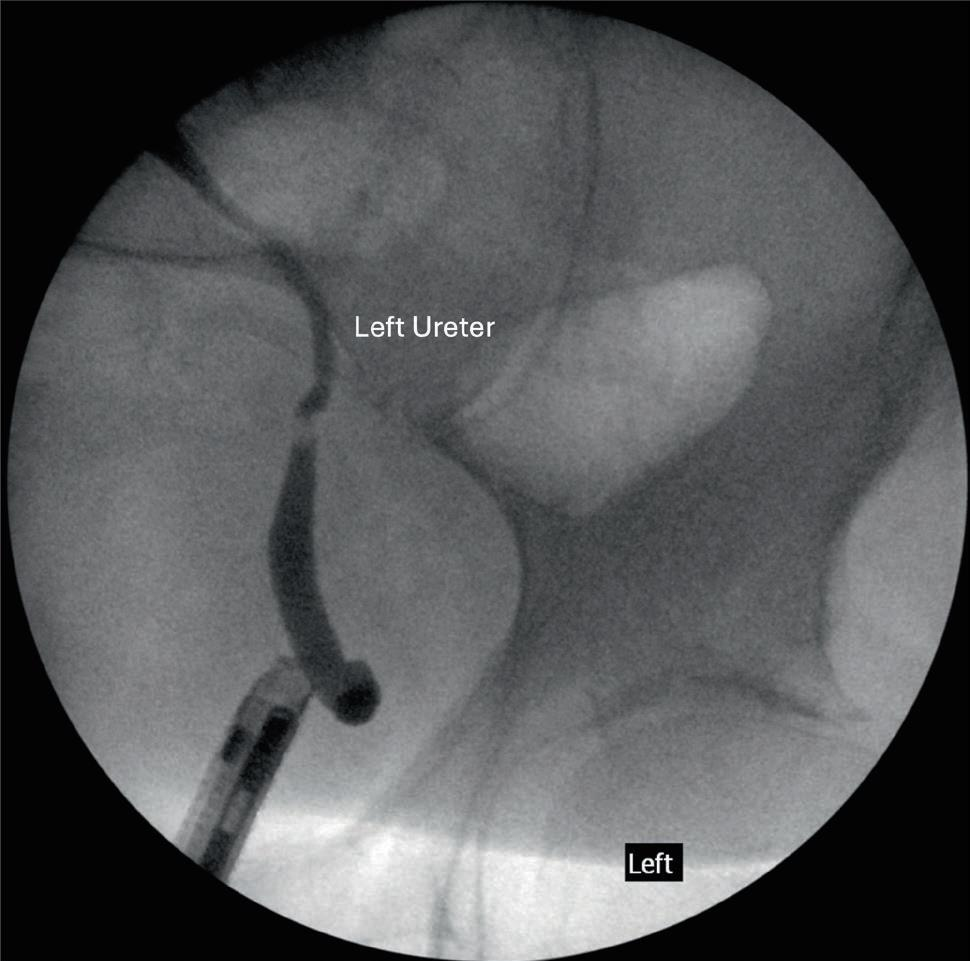
(a)

**Figure:**
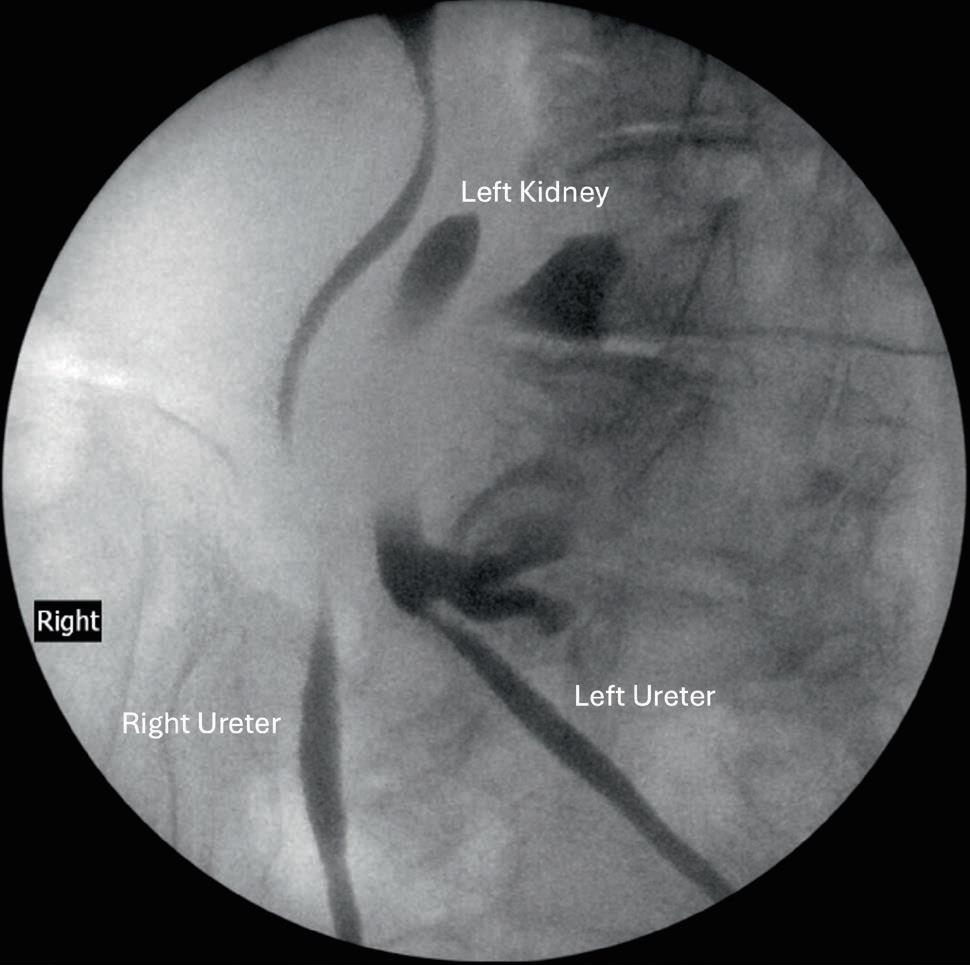
(b)

## 3. Discussion

During fetal development, as the metanephros ascends from the sacral level to the lumbar level, the blood supply ascends with it. New arteries extend from the aorta cranially, and caudal arteries regress as the metanephros migrates cranially [4]. If the cranial migration of the metanephros prematurely stops, then the resulting renal perfusion will be irregular. This unusual anatomy must be understood before surgery or radiation treatment in the lower abdominal or pelvic regions. CT‐urogram is usually the preferred study to visualize the vasculature. The unfused crossed ectopic kidneys usually have highly anomalous vasculature. This individual′s crossed ectopic kidney and arterial and venous blood supply were cranial to those of some other recent case reports describing this abnormality [2, 6, 7]. The position of the ectopic kidney high in the false pelvis benefited the patient as it did not limit his planned chemoradiation. Interestingly, the patient′s lower poles of both kidneys were supplied by branches from the same aberrant artery originating from the abdominal aorta. Based on the author′s literature search, this irregularity in the vasculature is exceedingly rare and has only been shown in one other case report of unfused crossed renal ectopia [2].

## Funding

No funding was received for this manuscript.

## Disclosure

All authors have read and approved the final version of the manuscript. Adam J. Ferguson had full access to all of the data in this study and takes complete responsibility for the integrity of the data and the accuracy of the data analysis.

## Ethics Statement

All participants submitted written approval of informed consent to be included in this case report.

## Conflicts of Interest

The authors declare no conflicts of interest.

## Data Availability

Data sharing is not applicable to this article as no datasets were generated or analyzed during the current study.
